# Molecular mechanisms of *s*-methoprene-induced growth inhibition in *Ephestia elutella* (Hübner) (Lepidoptera: Pyralidae): insights from transcriptomic analysis

**DOI:** 10.1093/jisesa/ieaf035

**Published:** 2025-06-10

**Authors:** Chao Huang, Jianhua Lü, Chunqi Bai, Yafei Guo, Chao Guo, Jizhen Song, Jiaqin Xi

**Affiliations:** Henan Collaborative Innovation Center for Grain Storage Security, School of Food and Strategic Reserves, Henan University of Technology, Zhengzhou, China; Henan Collaborative Innovation Center for Grain Storage Security, School of Food and Strategic Reserves, Henan University of Technology, Zhengzhou, China; Henan Collaborative Innovation Center for Grain Storage Security, School of Food and Strategic Reserves, Henan University of Technology, Zhengzhou, China; Henan Collaborative Innovation Center for Grain Storage Security, School of Food and Strategic Reserves, Henan University of Technology, Zhengzhou, China; Laboratory of Grain Storage and Pest Control, Grain Storage and Logistics National Engineering Laboratory, Guangdong Institute for Cereal Science Research, Guangzhou, China; Zhengzhou Tobacco Research Institute of CNTC, Zhengzhou, China; Zhengzhou Tobacco Research Institute of CNTC, Zhengzhou, China

**Keywords:** *s*-Methoprene, juvenile hormone analog, *Ephestia elutella*, molecular mechanisms, transcriptome analysis, RT-qPCR validation

## Abstract

*Ephestia elutella* is a globally distributed storage pest, and its growth and development are regulated by juvenile hormones. To investigate the molecular mechanisms underlying the response of *E. elutella* larvae to the juvenile hormone analog *s*-methoprene, this study examined the effects of *s*-methoprene on the growth and development of *E. elutella*, explored the response of *E. elutella* to *s*-methoprene exposure by transcriptomic analysis, and confirmed its hub genes by RT-qPCR experiments. Larval mortality of *E. elutella* increased and adult emergence decreased with increasing exposure durations and doses of *s*-methoprene. After exposure at 5 × 10⁻⁵ mg/cm² of *s*-methoprene for 4 wk, a few of larvae pupated, but failed to emerge into adults, while at 50 × 10⁻⁵ mg/cm² for 4 wk, larvae were completely unable to pupate. Transcriptomic analysis identified 2,569 and 6,719 differentially expressed genes in the EE0 vs. EE5 and EE0 vs. EE50, respectively. Weighted Gene Co-expression Network Analysis identified 5 modules, with the yellow module most relevant to EE5. The genes in the yellow module were significantly enriched in biological processes. The Cluster-6182.18691, Cluster-6182.8343, Cluster-6182.28346, and Cluster-6182.21392 were hub genes in the yellow module. *s*-Methoprene directly or indirectly inhibited the growth and development of *E. elutella* larvae by affecting critical biological processes, such as hormonal regulation, etc. RT-qPCR validation confirmed the reliability of the transcriptomic data. This study provides important foundational data and theoretical insights into the molecular mechanisms of *E. elutella* in response to *s*-methoprene.

## Graphical Abstract

**Graphical Abstract:**
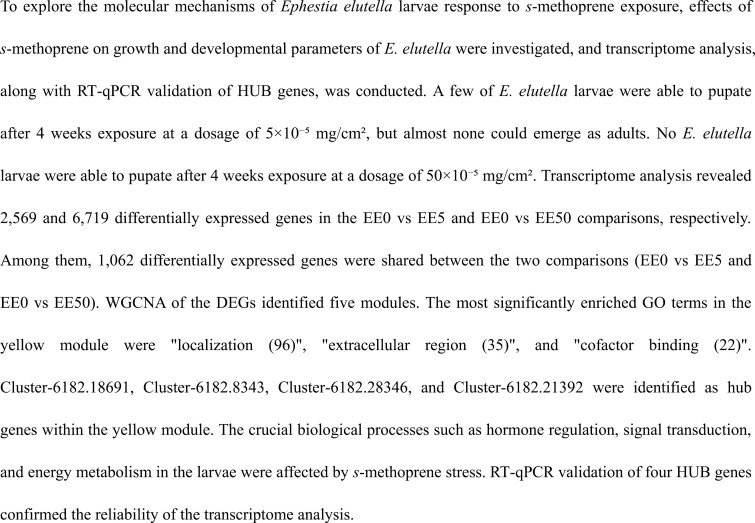

*Ephestia elutella* (Hübner) (Lepidoptera: Pyralidae) is a significant storage pest insect, and it is widely distributed across the world (Ashworth 1993). The larvae spin silk to bind tobacco leaves together, boring into them and causing holes, sometimes leaving only the leaf veins (Rumbos et al. 2018). Their excrement, cast skins, and dead bodies can lead to mold growth on the leaves, producing an unpleasant odor and severely contaminating the tobacco, thereby lowering both its grade and quality of cigarettes (Trematerra 2020). Currently, the primary methods for controlling *E. elutella* include applying chemical agents such as phosphine, sulfuryl fluoride, and deltamethrin. Although these methods are simple and effective, their prolonged and extensive use has led to many negative issues such as environmental pollution, chemical residues, and increasing pest resistance (Bell 1992, Baltaci et al. 2009, Athanassiou et al. 2022).

Insect growth regulators (IGRs) are a class of specific insecticides that cause insect death by disrupting their normal development. They are characterized by their low toxicity, high selectivity, and environmental safety. Based on their modes of action and structural types, IGRs are mainly classified into juvenile hormone (JH) analogs, chitin synthesis inhibitors, and ecdysteroid analogs (Mondal and Parween 2000, Tunaz and Uygun 2004). Among these, JH analogs are commonly used as insecticides in practice and are frequently employed in experimental research to regulate the growth and development of target insects. JH analogs can control embryonic development in insects and affect the growth and development of insect larvae (Riddiford and Williams 1967, Schal et al. 1997). *s*-Methoprene, a JH analog, was developed in 1970s. It mimics the naturally occurring JHs in insects, disrupting their normal developmental processes and inhibiting their metamorphosis from larvae to adults (Henrick 2007). This mechanism makes *s*-methoprene effective against a variety of pests, particularly in grain storage and public health, where it is used to control fleas, mosquitoes, and other stored-product pests (Lawler 2017, Wijayaratne et al. 2018).

With the rapid advancement and application of high-throughput sequencing technology, transcriptome sequencing has been increasingly utilized in the field of insect toxicology, which provides essential support for research on pests that lack reference genomes. In recent years, researchers have utilized transcriptome sequencing to study the metabolism, detoxification, and resistance mechanisms of various pests, including sanitary pests such as *Anopheles gambiae*, field crop pests like *Laodelphax striatellus*, and stored-product pests like *Tribolium castaneum*. These studies have explored the resistance mechanisms to various insecticides, including phosphine, pyrethroids, and chlorpyrifos (Bonizzoni et al. 2012, Zhang et al. 2012, Kim et al. 2023).

Previous studies have shown that *s*-methoprene exhibits certain control effects on *E. elutella*, but the specific mechanisms underlying its action remain unclear. Elucidating the molecular mechanisms of *E. elutella* response to *s*-methoprene is crucial for developing efficient and sustainable management strategies. Thus, we conducted transcriptomic sequencing and bioinformatics analysis to systematically compare gene expression differences in *E. elutella* larvae before and after *s*-methoprene exposure. Key differentially expressed genes (DEGs) were further validated using real-time quantitative PCR (RT-qPCR).

## Materials and Methods

### Insects and *s*-Methoprene Treatment

The *E. elutella* was obtained from the Stored-Grain Pest Laboratory at Henan University of Technology and has been maintained for more than 3 generations. The rearing conditions were 28 ± 2 °C, relative humidity of 75 ± 5%, with a diet consisting of wheat bran, soybean powder, honey, glycerol, and yeast in a mass ratio of 9:8:1:1:1. Adults were placed in 5-liter plastic containers under a 0:24 light–dark cycle (complete darkness). To obtain 15-d-old larvae, approximately 500 adults were transferred to a plastic container, which was sealed with a 20-mesh screen, and inverted over a petri dish for mating and egg-laying for 24 h. The eggs were then transferred to glass bottles containing the diet and reared under the above conditions for 20 d (including egg incubation time). The larvae were then collected for the experiment.

Thirty 15-d-old *E. elutella* larvae were exposed to filter paper treated at 0.0, 5.0, and 50.0 × 10⁻⁵ mg/cm² of *s*-methoprene for 4 wk, and the *E. elutella* larvae were referred as EE0, EE5, and EE50 in the study, respectively. The filter papers were firmly coated at the bottom of a petri dish (11 cm inner diameter and 3 cm high). The sides of the petri dish were coated with polytetrafluoroethylene to prevent the larvae from escaping. In each petri dish, 0.5 g of mixed feed and 30 *E. elutella* larvae were introduced. The dishes were then incubated in a biochemical incubator at 28 ± 2 °C, 75 ± 5% relative humidity, and complete darkness. During the experiment, shed skins and frass were removed every 7 d, 0.5 g of fresh feed was added, and the number of larvae, pupation rate, and mortality rate were recorded. The experiment was repeated 3 times. Data were analyzed using ANOVA to assess overall differences. Tukey’s test was then performed for pairwise comparisons, with all analyses conducted in IBM SPSS Statistics 26.

### RNA Isolation and Transcription Sequencing

After continuous exposure of *E. elutella* larvae to different doses of *s*-methoprene (0.0, 5.0, and 50.0 × 10⁻⁵ mg/cm²) for 4 wk, the surviving larvae were first frozen in liquid nitrogen, and then stored in an ultra-low temperature freezer at −80 °C, which were referred as EE0, EE5, and EE50, respectively, in triplicates. The frozen *E. elutella* larvae were ground into a fine powder, and RNA was extracted using Trizol (Invitrogen, USA). The concentration and purity of the extracted RNA were measured using a Nanodrop2100 bioanalyzer, and RNA integrity was checked via agarose gel electrophoresis.

The RNA was then fragmented under alkaline conditions. Following fragmentation, first-strand cDNA was synthesized using random hexamer primers, and second-strand cDNA synthesis was subsequently performed. After end repair, addition of an A-tail, adaptor ligation, fragment selection, amplification, and purification, the library was prepared. The constructed library was initially quantified using a Qubit 2.0 Fluorometer, and the insert size was assessed using an Agilent 2100 bioanalyzer. Once the insert size met expectations, the library’s effective concentration was precisely quantified using RT-qPCR (with an effective concentration greater than 1.5 nM) to ensure library quality.

After confirming the library’s quality, different libraries were pooled based on their effective concentration and the desired sequencing data output, and then subjected to Illumina sequencing. The raw sequencing data obtained included a small number of reads with sequencing adaptors or low-quality reads. To ensure the quality and reliability of the data analysis, the raw data were filtered as follows: (i) removal of reads containing adaptors; (ii) removal of reads containing *N* (where *N* indicates an indeterminate base); and (iii) removal of low-quality reads (reads where more than 50% of the bases have a Qphred score ≤ 20).

### Sequence Assembly, Annotation, and Bioinformatic Analysis

Trinity (Grabherr et al. 2011) were used for transcriptome assembly of clean reads. First, all fastq format reads (including single-end and paired-end reads) were loaded. Then, Trinity used the de Bruijn graph algorithm to break the reads into k-mers (short fragments with a length of k bp), counted the types and frequencies of the k-mers, and selected the k-mer with the highest frequency as the initial seed. The system then extended from the seed in the 3′ direction, and after each extension, the extension path was determined based on the k-mer with the highest frequency, continuing until extension was no longer possible. By establishing connections between k-mers, a de Bruijn graph was constructed, and based on the overlapping information in the graph, k-mers were assembled into contig sequences. Contigs with similar regions (greater than k − 1-mers) were clustered to form components, and different de Bruijn graphs were constructed for each component. Next, the reads and paired-end information were used for alignment verification. During the assembly process, Trinity’s Butterfly module simplified the de Bruijn graph into linear sequences, handled alternative splicing isoforms, and eliminated erroneous assembly paths, ultimately generating complete transcript sequences. Upon completion of the assembly, the results were saved in the output file TRINITY.fasta, which contained all reconstructed full-length transcripts.

To obtain comprehensive gene function information, gene function annotation was performed using 7 major databases, including Nr, Nt, PFAM (Sammut et al. 2008), KOG/COG, Swiss-prot, KO, and GO.

The transcriptome assembled by Trinity was used as the reference sequence (Ref), and the clean reads from each sample were mapped onto the Ref. Reads with mapping quality scores below 10, unpaired reads, and reads that mapped to multiple regions of the genome were filtered out. The mapping process utilized RSEM software, with Bowtie2 as the alignment tool. In RSEM, the mismatch parameter was set to 0, while other parameters used Bowtie2’s default settings. The number of reads mapped to a gene was referred to as the read count, and the read counts were converted to Fragments Per Kilobase of transcript per Million mapped reads (FPKM) values to calculate gene expression levels.

DEGs were identified using DESeq2 software with criteria of *P*_*adj*_ < 0.05 and |log2(Fold Change)| > 1. Gene Ontology (GO) enrichment analysis of DEGs was conducted using the GOseq (v3.4.4) R package. Kyoto Encyclopedia of Genes and Genomes (KEGG) enrichment analysis of DEGs was performed using the KOBAS (Mao et al. 2005) software package.

### Weighted Correlation Network Analysis

To explore whether there were common expression patterns among genes in different samples, Weighted Gene Co-expression Network Analysis (WGCNA) was used to identify and cluster genes with similar expression patterns, and analyze the association between modules and specific traits or phenotypes. The Pearson correlation coefficient between any 2 genes was calculated. The soft threshold for constructing the co-expression network was determined based on the scale-free topology fit index. The entire network was clustered using Topological Overlap Matrix, grouping genes with high connectivity into modules. To define the branches of the modules, the dynamic tree cut algorithm was used to cut the hierarchical clustering tree. Cytoscape (v3.7.1) was employed for network visualization of highly connected modules with specific WGCNA edge weights (Saito et al. 2012).

### RT-qPCR Confirmation

Four hub genes were selected for RT-qPCR to verify the reliability of the RNA-Seq results. Primers were designed using Primer Premier 5.0 and synthesized by Sangon Biotech (Shanghai, China). RNA quality and concentration were assessed using NanoDrop 2000, and 1 μg of RNA was used for cDNA synthesis with the Maxima Reverse Transcriptase Kit. The RT-qPCR experiments were conducted using 2× SG Fast qPCR Master Mix (High Rox) with 3 biological replicates, each from independent samples, for each gene. The relative expression levels of each gene were calculated using the 2^−∆∆Ct^ method (Livak and Schmittgen 2001). Beta-actin (GenBank: MW718150) was used as the reference gene (Table 1). Data were first analyzed using ANOVA to assess overall differences, followed by Tukey’s test for pairwise comparisons of significant results, which were conducted in IBM SPSS Statistics 26. Finally, linear regression analysis was performed to assess the correlation between RNA-Seq and RT-qPCR gene expression changes. Three replicates were conducted.

**Table 1.** RT-qPCR primers

Gene ID	Primer	Annealing temperature (°C)	ProdSize (bp)
Beta-actin-F1	GTGCCCATCTATGAAGGTTACG	59.1	154
Beta-actin-R1	CCTTGATGTCCCTCACGATTT	58.9	
Cluster-6182.21392-F	GAGAATGGGTCCGCAACTG	58.3	134
Cluster-6182.21392-R	CATACAGATTACGCCGTGAGG	58.3	
Cluster-6182.8343-F	CAAGCACGAACTGTAGGATCAA	58.1	115
Cluster-6182.8343-R	TTGGCTGTGTAGGAAACCGA	58.9	
Cluster-6182.28346-F	CGTCCCGCTTCCGATTTA	59.3	244
Cluster-6182.28346-R	ATGCGTTGTGCCACGATACT	58.8	
Cluster-6182.18691-F	CTCCCAAAGGAATCCCACC	58.9	171
Cluster-6182.18691-R	ATCCCCTAGATGAACCAGCAA	58.7	
Cluster-6182.18691-F	CTCCCAAAGGAATCCCACC	58.9	171
Cluster-6182.18691-R	ATCCCCTAGATGAACCAGCAA	58.7	

## Results

### Effects of *s*-Methoprene Treatment on Growth and Development Parameters of *E. elutella*

The dose of *s*-methoprene had a significant impact on the growth and development of *E. elutella* (Table 2). After 2 weeks of continuous exposure to *s*-methoprene, there was no significant difference in larval survival or mortality rates between the control group and the treatment groups. However, at a dose of 50.0 × 10⁻⁵ mg/cm², the larval mortality rate was significantly higher, and the pupal survival rate was significantly lower compared to other doses (*F* = 8.81; df = 2, 6; *P* = 0.016; *F* = 16.29; df = 2, 6; *P* = 0.004). After 3 wk of continuous exposure, the larval survival rate in the control group was significantly lower, while the pupal survival and adult emergence rates were significantly higher compared to the treatment groups (*F* = 36.71; df = 2, 6; *P* < 0.001; *F* = 268.00; df = 2, 6; *P* < 0.001; *F* = 29.72; df = 2, 6; *P* = 0.001). Following 4 wk of continuous exposure, the larval survival and mortality rates in the treatment groups were significantly higher than those in the control group (*F* = 16.00; df = 2, 6; *P* = 0.004; *F* = 47.81; df = 2, 6; *P* < 0.001). Conversely, the adult emergence rate in the control group was significantly higher than that in the treatment groups (*F* = 439.29; df = 2, 6; *P* < 0.001).

**Table 2.** Effects of different doses of *s*-methoprene treatment on the growth and development parameters of *Ephestia elutella* larvae

Time (Wk)	Growth and development parameters (%)	Dose (× 10^−5^ mg/cm^2^)		
		0	5	50
1	Larval survival	100.00 ± 0.00a	97.78 ± 2.22a	95.55 ± 2.22a
	Larval mortality	0.00 ± 0.00a	2.22 ± 2.22a	4.45 ± 2.22a
	Pupal survival	…	…	…
	Pupal mortality	…	…	…
	Pupal eclosion	…	…	…
2	Larval survival	64.45 ± 7.78a	70.00 ± 5.09a	81.11 ± 4.84a
	Larval mortality	0.00 ± 0.00b	5.56 ± 2.94b	18.89 ± 4.84a
	Pupal survival	36.67 ± 6.94a	24.45 ± 4.01a	0.00 ± 0.00b
	Pupal mortality	…	…	…
	Pupal eclosion	…	…	…
3	Larval survival	18.89 ± 2.94b	47.78 ± 2.94a	57.78 ± 4.01a
	Larval mortality	0.00 ± 0.00b	12.22 ± 4.01b	42.22 ± 4.01a
	Pupal survival	60.00 ± 0.00a	13.33 ± 3.33b	0.00 ± 0.00c
	Pupal mortality	0.00 ± 0.00b	24.45 ± 2.22a	0.00 ± 0.00b
	Pupal eclosion	21.11 ± 2.94a	2.22 ± 2.22b	0.00 ± 0.00b
4	Larval survival	0.00 ± 0.00b	17.78 ± 11.28b	53.33 ± 3.33a
	Larval mortality	0.00 ± 0.00c	18.89 ± 4.84b	46.67 ± 3.33a
	Pupal survival	6.67 ± 3.85ab	15.56 ± 5.88a	0.00 ± 0.00b
	Pupal mortality	0.00 ± 0.00b	41.11 ± 5.56a	0.00 ± 0.00b
	Pupal eclosion	93.33 ± 3.85a	6.67 ± 1.93b	0.00 ± 0.00b

*Note:* ‘…’ indicates no data available. The data in the table represent the rates of growth and development parameters, expressed as mean ± standard error. Within a row, different lowercase letters indicate significant differences between different doses of *s*-methoprene (*P* < 0.05).

### Sequencing Data Analysis

**Fig. 1.**
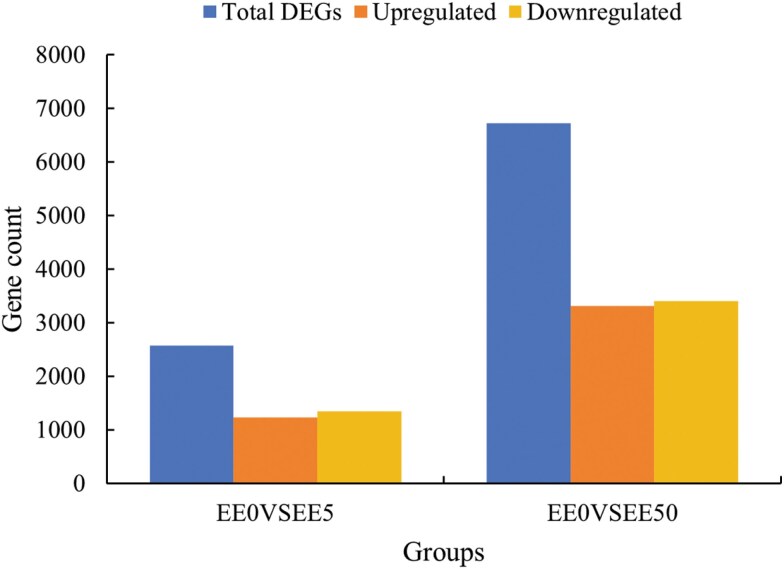
Statistical results of DEGs in *Ephestia elutella* larvae under *s*-methoprene exposure. *Note:* EE0, EE5, and EE50 represent the *E. elutella* larvae sample which were exposed to 0.0, 5.0, and 50.0 × 10⁻⁵ mg/cm² of *s*-methoprene for 4 wk, respectively. The same as Figs. 2 to 4 and Fig. 6.

**Fig. 2.**
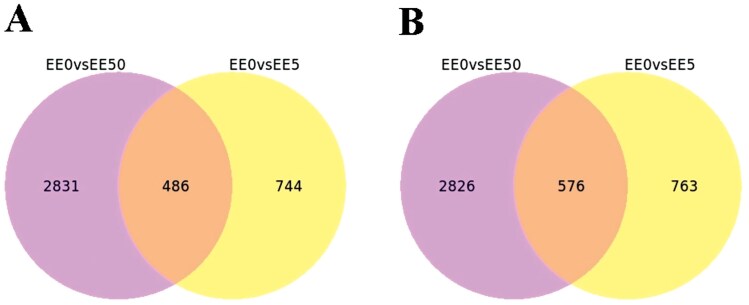
Venn diagrams of differentially co-expressed genes in *Ephestia elutella* larvae under *s*-methoprene exposure. A) Upregulated genes, B) downregulated genes.

After sequencing, low-quality sequences were removed from the 12 samples. The number of Clean reads for these samples ranged between 21.9 and 23.6 million. The Q20 and Q30 values were both above 98% and 94%, respectively, and the base error rate was 0.01% (Table 3). Functional annotation of the transcripts was carried out to obtain gene function information. For comprehensive gene function analysis, annotations were conducted using 7 major databases: Nr, Nt, PFAM (Sammut et al., 2008), KOG/COG, Swiss-prot, KO, and GO. This process resulted in the annotation of 18,728, 30,006, 13,075, 6,538, 11,353, 8,389, and 13,075 genes, respectively (Table 4). Trinity software was used to assemble all Clean reads, followed by further processing and de-redundancy to produce Unigene sequences, resulting in a total of 40,176 genes. The average length of the Unigene sequences was 1,310 bp, with an N50 value of 2,259 (Table 5). DEGs were identified using DESeq2 software, with thresholds of *P*_*adj*_ < 0.05 and |log2(Fold Change)| > 1. A total of 8,043 DEGs were detected, including 4,061 upregulated and 4,165 downregulated genes. In the comparison between EE0 and EE5, 2,569 genes were identified, with 1,230 upregulated and 1,339 downregulated genes. In the comparison between EE0 and EE50, 6,719 genes were identified, with 3,317 upregulated and 3,402 downregulated genes (Fig. 1). Among the common DEGs identified between EE0 vs EE5 and EE0 vs EE50, there were 486 upregulated and 576 downregulated genes (Fig. 2).

**Table 3.** Sequencing quality report

Sample	Raw reads	Raw bases	Clean reads	Clean bases (G)	Error rate	Q20 (%)	Q30 (%)	GC pct
EE0_1_	23921023	7.18	23328804	7.00	0.01	98.65	96.23	46.29
EE0_2_	23497918	7.05	22839390	6.85	0.01	98.09	94.43	45.41
EE0_3_	22908566	6.87	22286613	6.69	0.01	98.20	94.73	44.96
EE5_1_	22862664	6.86	22127439	6.64	0.01	98.19	94.72	46.40
EE5_2_	23439535	7.03	22740873	6.82	0.01	98.10	94.45	46.52
EE5_3_	23597200	7.08	23037026	6.91	0.01	98.94	97.01	41.04
EE50_1_	22644813	6.79	22245966	6.67	0.01	98.68	96.28	47.50
EE50_2_	22618385	6.79	21915996	6.57	0.01	98.93	96.93	47.68
EE50_3_	24260218	7.28	23590563	7.08	0.01	98.05	94.33	47.08

*Note:* In this table, EE0, EE5, and EE50 represent samples treated with 0, 5, and 50 × 10⁻⁵ mg/cm² doses of *s*-methoprene, respectively. The subscripts 1, 2, and 3 indicate 3 replicate samples for each treatment. Q20 means the percentage of bases with a quality score greater than 20 among the total bases, where the quality score is a measure of base calling accuracy. A higher quality score indicates higher confidence in the base identification. Q30 indicates the percentage of bases with a quality score greater than 30, which reflects an even higher level of base calling accuracy. GC content refers to the percentage of bases comprised of G and C in the total base count.

**Table 4.** Gene annotation success rate statistics

Database	Number of unigenes	Percentage (%)
Annotated in NR	18,728	46.61
Annotated in NT	30,006	74.68
Annotated in KO	8,389	20.88
Annotated in Swiss-Prot	11,353	28.25
Annotated in PFAM	13,075	32.54
Annotated in GO	13,075	32.54
Annotated in KOG	6,538	16.27
Annotated in all databases	4,369	10.87
Annotated in at least one database	32,618	81.18
Total unigenes	40,176	100.00

**Table 5.** Distribution of assembly lengths

Type	Min length	Mean length	Median length	Max length	N50	N90	Total nucleotides
Transcript	301	1,534	974	28,425	2,459	637	156,166,519
Unigene	301	1,310	700	28,425	2,259	492	52,637,053

### GO and KEGG Pathway Enrichment for DEGs

**Fig. 3.**
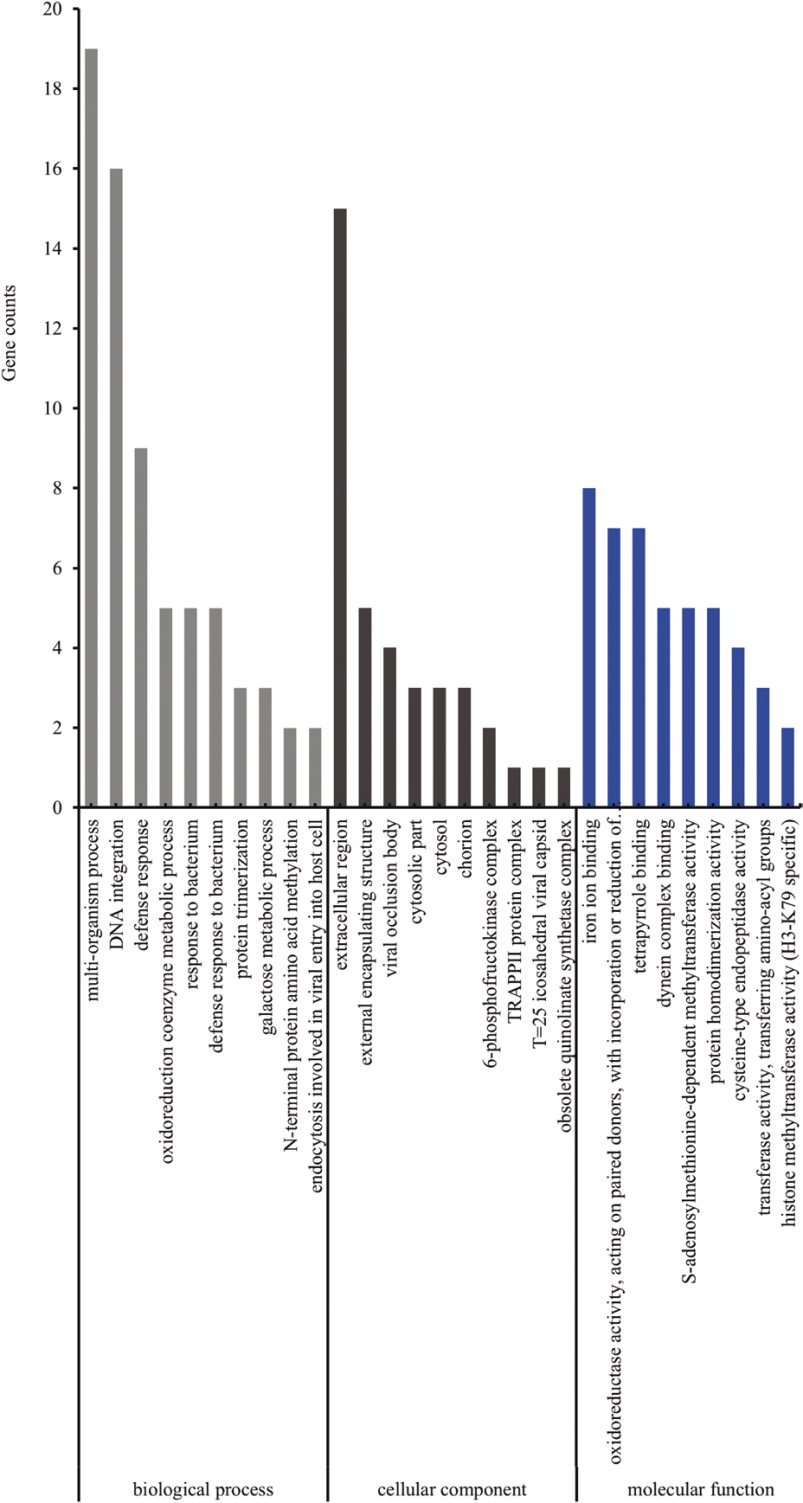
GO enrichment analysis of upregulated differentially co-expressed genes in *Ephestia elutella* EE0 vs EE5 and EE0 vs EE50 samples.

**Fig. 4.**
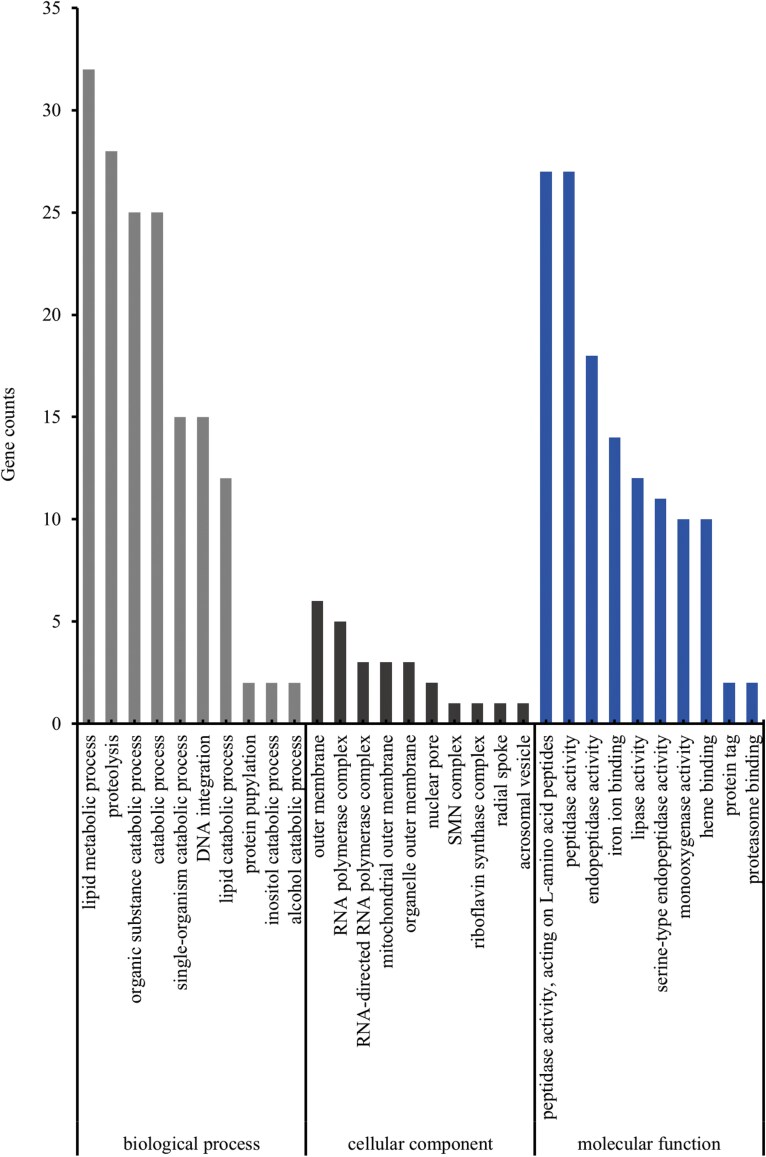
GO enrichment analysis of downregulated differentially co-expressed genes in *Ephestia elutella* EE0 vs EE5 and EE0 vs EE50 samples.

To understand the functional roles of these differentially co-expressed genes in biological processes, cellular components, and molecular functions, we performed GO functional enrichment analysis on the sets of 486 upregulated and 576 downregulated differentially co-expressed genes using the clusterProfiler software. The analysis revealed that 169 upregulated differentially co-expressed genes were annotated with GO terms, accounting for 34.77% of all upregulated differentially co-expressed genes. These annotated genes were categorized into 3 main categories: “Biological Process” (126 genes), “Cellular Component” (81 genes), and “Molecular Function” (138 genes). The most significantly enriched terms within these categories were “multi-organism process” (19 genes), “extracellular region” (15 genes), and “multi-organism process” (8 genes) (Fig. 3). For the downregulated differentially co-expressed genes, 237 were annotated with GO terms, representing 41.15% of all downregulated differentially co-expressed genes. These annotated genes were similarly categorized into “Biological Process” (175 genes), “Cellular Component” (97 genes), and “Molecular Function” (186 genes). The most significantly enriched terms in these categories were “lipid metabolic process” (32 genes), “outer membrane” (6 genes), and “peptidase activity, acting on L-amino acid peptides” (27 genes) (Fig. 4).

To understand the functions of these genes within *E. elutella* and the biological pathways they are involved in, we conducted KEGG pathway enrichment analysis on the DEGs. Using the KEGG database, 231 DEGs in the EE0 vs EE5 comparison were enriched in 203 pathways, accounting for 9.00% of the total DEGs. The top 2 most significantly enriched pathways in KEGG were: “Insect hormone biosynthesis” (10 genes), “Lysosome” (21 genes), and “Starch and sucrose metabolism” (15 genes) (Table 6). In the EE0 vs EE50 comparison, 984 DEGs were enriched in 279 pathways, accounting for 14.65% of the total DEGs. The top 3 most significantly enriched pathways in KEGG were: “Insect hormone biosynthesis” (10 genes), “Lysosome” (21 genes), and “Starch and sucrose metabolism” (15 genes) (Table 7).

**Table 6.** The top 10 most significantly enriched KEGG metabolic pathways in differential genes of EE0 vs EE5 samples

KEGG pathway	KEGG ID	Gene counts	Enrichment factor	*P*
Insect hormone biosynthesis	ko00981	10	168.4233172	9.65E−07
Lysosome	ko04142	21	53.82223397	2.02E−05
Starch and sucrose metabolism	ko00500	15	66.31668114	3.81E−05
AMPK signaling pathway	ko04152	18	56.84286955	4.13E−05
Biosynthesis of unsaturated fatty acids	ko01040	10	95.59161246	5.66E−05
Steroid biosynthesis	ko00100	6	101.0539903	1.40E−03
PPAR signaling pathway	ko03320	10	58.94816101	1.63E−03
Retinol metabolism	ko00830	9	60.06039047	2.45E−03
Fat digestion and absorption	ko04975	6	88.42224152	2.49E−03
Longevity regulating pathway—worm	ko04212	12	48.23031356	2.70E−03

**Table 7.** The top 10 most significantly enriched KEGG metabolic pathways in differential genes of EE0 vs EE50 samples

KEGG pathway	KEGG ID	Gene counts	Enrichment factor	*P*
Starch and sucrose metabolism	ko00500	38	168.0022589	2.11E−4
Proteasome	ko03050	25	200.9596398	3.43E−4
Glutathione metabolism	ko00480	32	174.1237987	4.00E−4
Glycolysis / Gluconeogenesis	ko00010	34	164.7318472	5.83E−4
Peroxisome	ko04146	42	147.07858	8.94E−4
PPAR signaling pathway	ko03320	29	170.9496669	9.15E−4
DNA replication	ko03030	20	191.1832249	2.04E−3
Hematopoietic cell lineage	ko04640	12	265.2667246	2.37E−3
Galactose metabolism	ko00052	18	192.9212542	3.11E−3
Base excision repair	ko03410	14	225.0747966	3.17E−3

### WGCNA Co-expression Network Analysis of DEGs

**Fig. 5.**
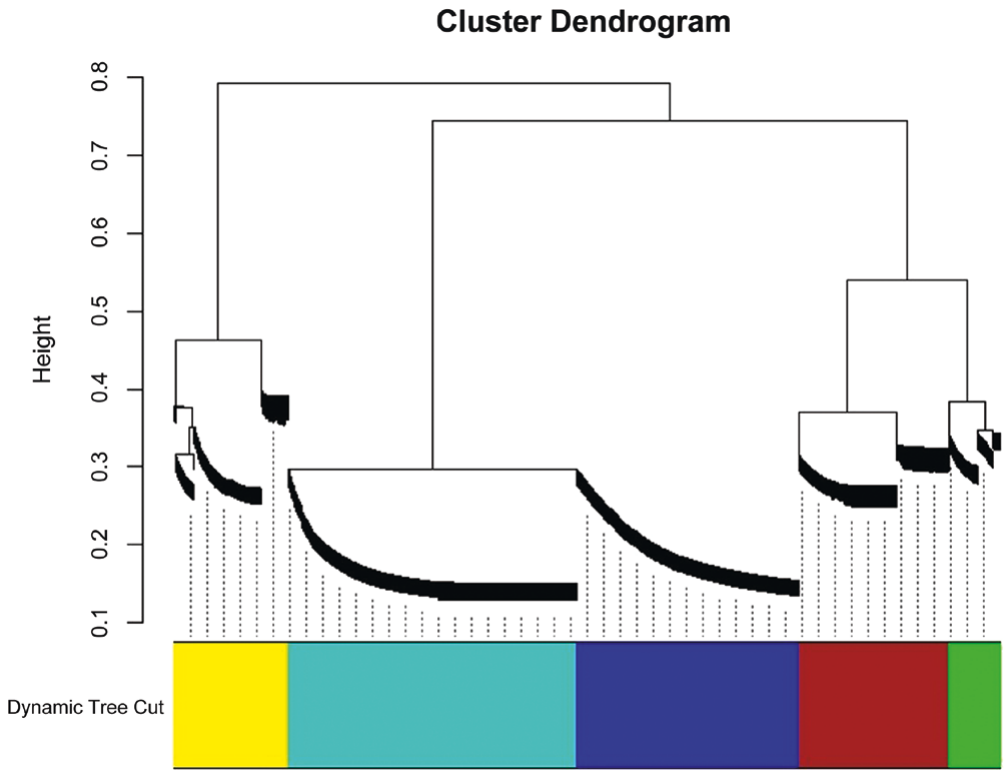
Gene clustering dendrogram and dynamic tree cutting results of the DEGs in *Ephestia elutella* samples. The dendrogram shows the clustering of genes based on their expression similarity, with the "Height" (vertical axis) representing the dissimilarity between clusters. The 5 co-expression modules are identified by dynamic tree cutting and represented by different colors at the bottom of the dendrogram.

**Fig. 6.**
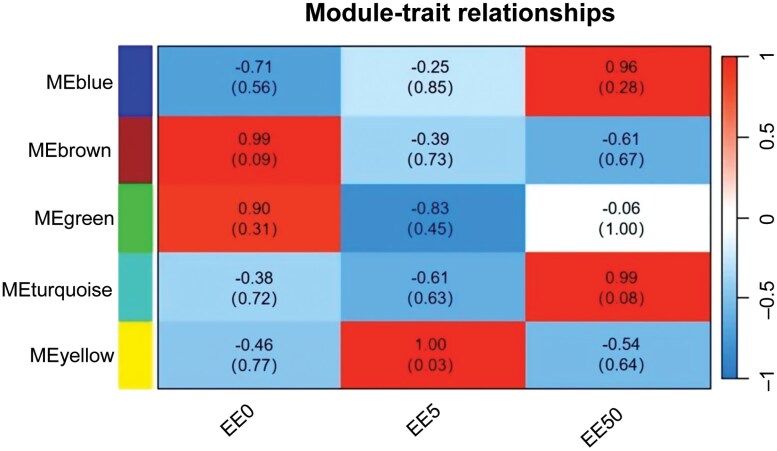
**Correlation analysis between gene modules and traits in** *Ephestia elutella* across different sample groups. Rectangles represent the correlation strength, ranging from −1 (strong negative correlation) to 1 (strong positive correlation), as indicated by the scale on the right vertical axis. Numbers in parentheses show the *P*-values for statistical significance (*P* < 0.05).

**Fig. 7.**
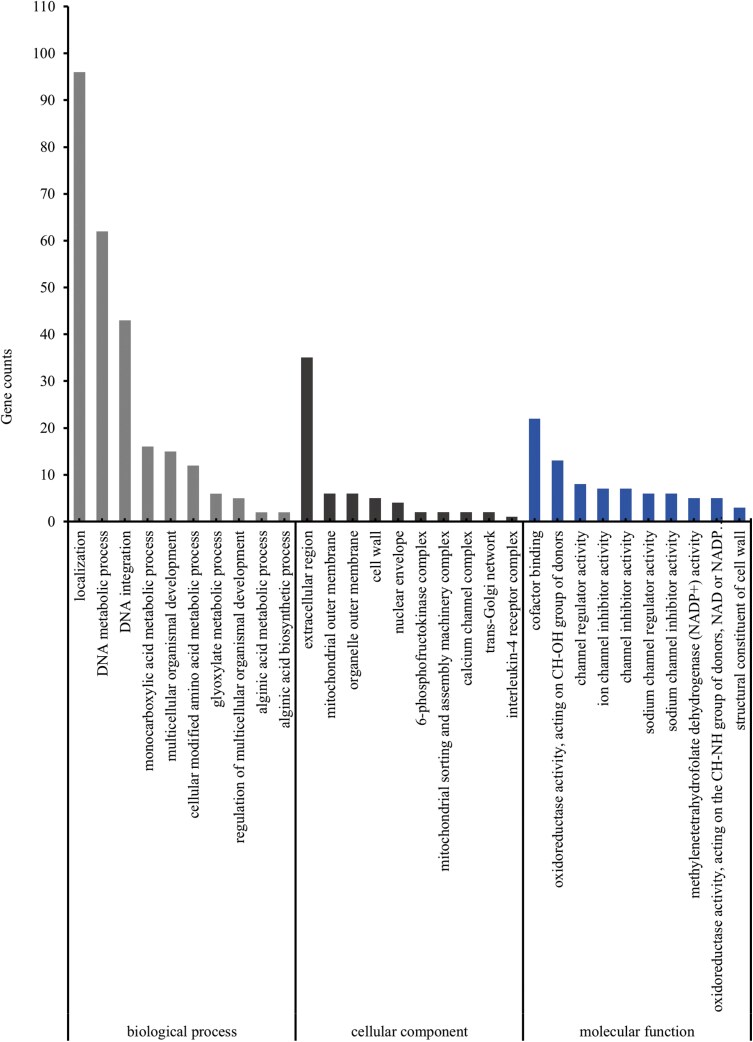
GO enrichment analysis of differentially co-expressed genes in the yellow module of *Ephestia elutella*.

**Fig. 8.**
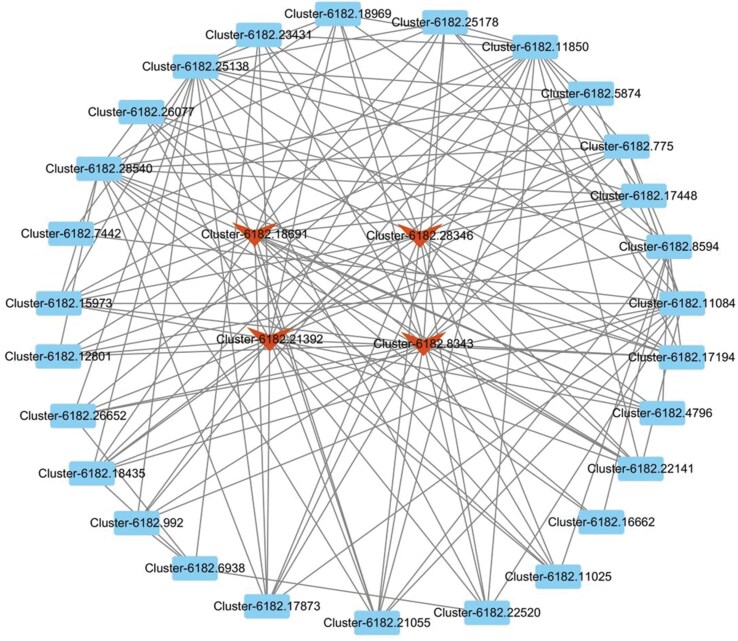
Interaction network of genes in the yellow module of *Ephestia elutella* with edge weights > 0.73.

To further understand the relationship between gene co-expression networks and gene modules with phenotypes, we constructed a weighted gene co-expression network for 8,043 differential genes, clustering into 5 modules (Fig. 5). We calculated the module eigengenes for these 5 modules and correlated them with the data from different *s*-methoprene treatments in *E. elutella* to identify modules significantly related to the phenotype. The yellow module showed a significant correlation with the EE5 sample (*P* ≦ 0.05) (Fig. 6). To reveal the functional characteristics of gene modules, we performed GO and KEGG enrichment analyses on the 1,108 genes in the yellow module. The GO enrichment analysis indicated that 462 genes were annotated, representing 41.70% of the yellow module genes, categorized into “Biological Process” (329 genes), “Cellular Component” (208 genes), and “Molecular Function” (335 genes). Based on *P-*values, the most significantly enriched categories in these 3 classes were: “DNA integration” (43), “extracellular region” (35), and “sodium channel regulator activity” (6) (Fig. 7). KEGG enrichment analysis revealed that 91 genes in the yellow module were enriched, accounting for 8.21% of the module. The top 3 most significantly enriched metabolic pathways based on *P*-values were: “Biosynthesis of unsaturated fatty acids” (6), “Longevity regulating pathway—worm” (8), and “Amebiasis” (5) (Table 8). Using a WGCNA edge weight threshold of >0.73 to screen the yellow module, we identified 4 genes as core nodes with the highest connectivity in the network: Cluster-6182.18691, Cluster-6182.8343, Cluster-6182.28346, and Cluster-6182.21392 (Fig. 8). These genes are referred to as hub genes.

**Table 8.** The top 10 most significantly enriched KEGG metabolic pathways in genes of the yellow module

KEGG pathway	KEGG ID	Gene counts	Enrichment factor	*P*
Biosynthesis of unsaturated fatty acids	ko01040	6	57.35496747	3.71E−4
Longevity regulating pathway—worm	ko04212	8	32.15354237	1.42E−3
Amoebiasis	ko05146	5	46.53802185	2.65E−3
PPAR signaling pathway	ko03320	5	29.47408051	1.53E−2
Insect hormone biosynthesis	ko00981	3	50.52699516	1.60E−2
Lysosome	ko04142	8	20.50370818	1.73E−2
ECM-receptor interaction	ko04512	3	42.44267593	2.43E−2
Terpenoid backbone biosynthesis	ko00900	3	39.29877401	2.91E−2
Type I diabetes mellitus	ko04940	2	64.30708474	3.33E−2
Peroxisome	ko04146	6	21.01122571	3.35E−2

### Validation of 4 Hub Genes by RT-qPCR

**Fig. 9.**
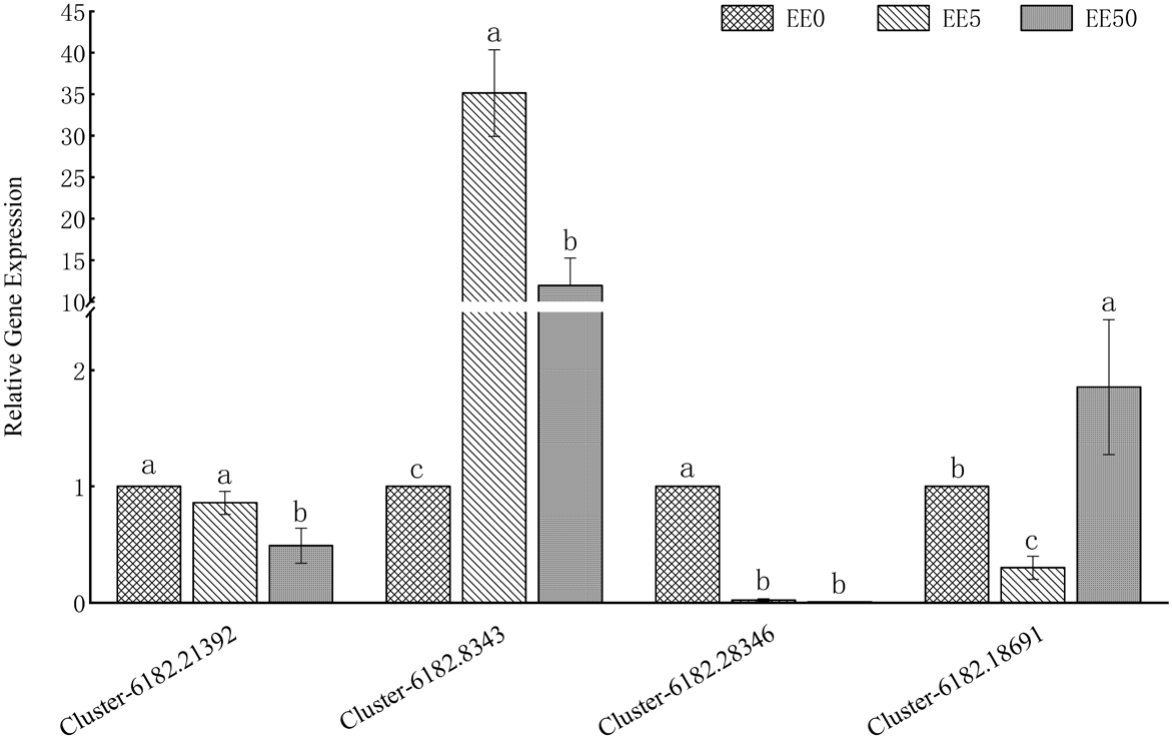
RT-qPCR analysis of the expression levels of 4 hub genes in *Ephestia elutella* EE0, EE5, and EE50 samples. The data in the figure represent the mean values of 3 replicates, and different lowercase letters above the bars indicate significant differences in gene expression among the different treatments for each individual gene (*P* < 0.05).

**Fig. 10.**
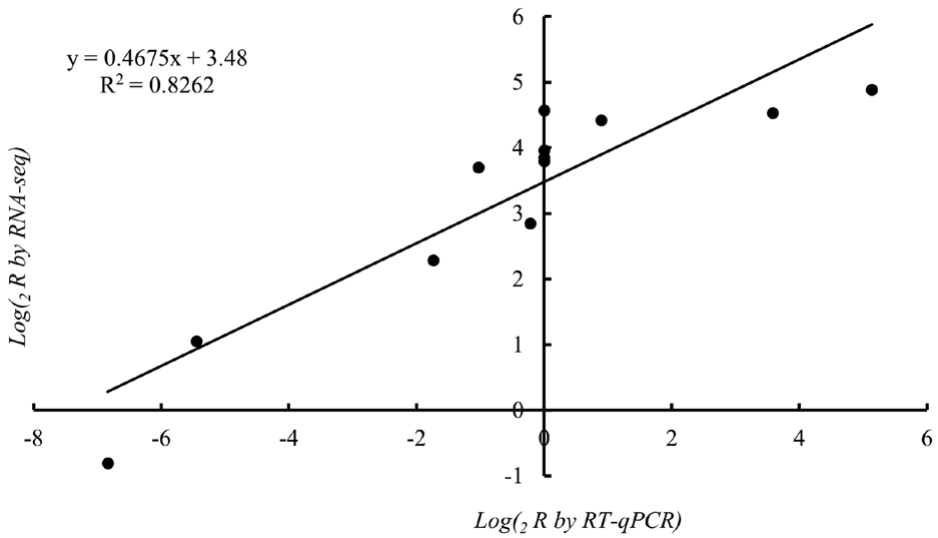
Correlation of gene expression levels measured by RNA-seq and RT-qPCR in *Ephestia elutella*. *R*² represents the correlation between gene expression changes measured by RT-qPCR (*x*-axis) and RNA-seq (*y*-axis).

To validate the accuracy of the RNA-Seq results, we selected 4 hub genes for RT-qPCR verification (Fig. 9). The result showed that the slope of the regression equation was 0.4675, indicating that RNA-Seq detected a larger magnitude of gene expression changes compared to RT-qPCR (Fig. 10). This difference may reflect the sensitivity variations between the 2 methods; RNA-Seq is more capable of capturing subtle expression changes, while RT-qPCR results are more conservative. However, the *R*² value of the regression analysis was 0.8262, demonstrating a strong correlation between the 2 methods. Despite the differences in the magnitude of changes, both methods are consistent in overall trends. Therefore, the results of this study remain reliable.

## Discussion

*Ephestia elutella* is an important global pest of stored products. Its larvae feed on tobacco leaves, leaving behind substantial debris and brown frass, which significantly affects both the commercial value and quality of the stored products (Ashworth 1993, Deng et al. 2018). The long-term and extensive use of traditional chemical insecticides has led to an increasing resistance of *E. elutella*, prompting the search for selective and environmentally friendly alternatives (Champ 1985, Baltaci et al. 2009). JH is essential for insect growth and development, involved in processes such as body morphology, molting, diapause, and the stimulation of yolk formation in female adults (Sugahara et al. 2017). In recent years, many researchers have investigated the effects of JH analogs (such as *s*-methoprene) on stored product pests (Wijayaratne et al. 2018), but there is limited research on the control of *E. elutella* larvae using *s*-methoprene. This study showed that *s*-methoprene effectively inhibited the growth and development of *E. elutella*. Significant differences were observed in growth and development parameters of larvae exposed to *s*-methoprene for different durations compared to the control group. Low doses of *s*-methoprene inhibited pupation, while high doses of *s*-methoprene inhibited pupal development, which was also validated in experiments of *E. cautella* with exposed to *s*-methoprene (Chandra and Tiwari 2013). To further investigate the molecular mechanisms of *E. elutella* response to *s*-methoprene exposure, this study used the Illumina sequencing platform to perform transcriptome sequencing of *E. elutella* larvae exposed to different doses of *s*-methoprene, identifying 9,288 significantly DEGs, including 4,547 significantly upregulated genes and 4,741 significantly downregulated genes.

In insects, the insulin signaling pathway primarily affects growth and development through metabolic regulation and energy balance. For instance, RNA interference (RNAi) targeting the insulin receptor in *Spodoptera frugiperda* can significantly reduce the concentration of JH III (Pan et al. 2022). RNAi-mediated silencing of the acid methyltransferase or methoprene-tolerant genes can reduce JH synthesis or its effects, and lower the expression of insulin-like peptide encoding genes (Sheng et al. 2011). This suggests that there is an interaction between JH and the insulin signaling pathway, with JH potentially regulating this pathway positively. The loss of the corpora allata (CA), which is the organ responsible for JH synthesis, leads to reduced levels of insulin signaling pathway activity, decrease PI3K-Akt signaling pathway activity, and negative regulation of Forkhead box O (FOXO) by the insulin signaling pathway, resulting in elevated FOXO activity levels in larvae; larvae lacking CA show reduced growth rates and smaller pupal sizes compared to controls (Mirth et al. 2014). However, in this study, we observed both upregulation and downregulation of genes involved in the “insulin signaling pathway”, “insulin secretion”, “insulin resistance”, “FOXO signaling pathway”, and “PI3K-Akt signaling pathway” in the EE0 vs EE5 and EE0 vs EE50 samples. This may reflect a complex regulatory mechanism of insulin signaling pathway genes under the influence of the exogenous JH analogue (*s*-methoprene) and endogenous JH conditions. This complexity might be achieved by slowing cell proliferation, promoting apoptosis, or inhibiting the expression of key developmental regulatory genes. Additionally, in the EE0 vs EE50 samples, we observed downregulation of 5 genes involved in the mTOR pathway downstream of the PI3K-Akt signaling pathway. This could directly inhibit cellular growth, proliferation, and metabolic functions in *E. elutella* larvae, leading to increased autophagy and decreased protein synthesis, which may be one of the reasons for the suppression of pupation and eclosion in *E. elutella*.

Insect molting hormone is a class of steroid hormones with potent molting activity, primarily existing in the form of 20-hydroxyecdysone (20E). GPCRs are membrane proteins characterized by 7 transmembrane domains. Ecdysone-responsible GPCR (Er GPCR) proteins transmit the 20E signal through the cell membrane, triggering a cascade of reactions that regulate the molting process in insects. Er GPCR-induced signaling modulates the release of intracellular Ca^2+^ from the endoplasmic reticulum and the influx of extracellular Ca^2+^, rapidly increasing cytoplasmic Ca^2+^ levels, which leads to protein phosphorylation and nuclear translocation (Cai et al. 2014, Zhao 2020). Through the activation of GPCR and Ca^2+^ signaling, 20E further triggers the phosphorylation and nuclear translocation of calcium/calmodulin-dependent protein kinase II (CaMKII), regulating USP1 lysine acetylation and forming the EcRB1/USP1 complex responsible for the transcription of 20E-responsive genes (Jing et al. 2015). This demonstrates that Ca^2+^, as a second messenger in intracellular signal transduction, plays a vital role in regulating physiological activities mediated by ecdysone in insects. In addition, a class of GPCRs exists in insects that binds both dopamine and 20E, known as the dopamine receptor DopEcR (Abrieux et al. 2014). During the larval stage, DopEcR functions as a dopamine receptor promoting larval feeding. Knockdown of the DopEcR gene expression inhibits feeding, growth, and pupation during the larval stage. 20E competes with dopamine for binding to DopEcR, inhibiting feeding and promoting the transition to pupation, while also transmitting the 20E signal via DopEcR. DopEcR-mediated 20E signaling can also induce an increase in intracellular cAMP levels, thereby activating the PKA pathway (Kang et al. 2019). Furthermore, 20E binding to the Gαq site of DopEcR increases intracellular Ca^2+^ levels, which subsequently activates the Protein kinase C** (**PKC) pathway (Elmogy et al. 2004). The dual regulatory functions of DopEcR on larval feeding and the 20E signaling pathway jointly promote the completion of insect metamorphosis. Thus, 20E regulates gene transcription and insect molting and metamorphosis by activating the GPCR/Ca^2+^ and GPCR/DopEcR/cAMP/PKA signaling pathways, forming the EcRB1/USP1 transcription complex in the nucleus. In this study, several downregulated genes related to Ca^2+^ and DopEcR were annotated in the EE0 vs EE5 comparison, such as “regulation of calcium ion transmembrane transport”, “calcium ion transmembrane transport”, “calcium ion import”, “calcium ion binding”, and “dopamine receptor signaling pathway”. This indicates that the slow growth and development of *E. elutella* larvae under *s*-methoprene treatment may be associated with the downregulation of genes involved in Ca^2+^ signaling pathways and DopEcR expression, leading to suppressed feeding and impaired 20E signal transduction.

In insects, molting and metamorphosis are coordinately regulated by ecdysteroid 20E and JH. Both 20E and JH play significant roles in regulating insect energy metabolism. In the process from larva to pupa in fruit flies, genome-wide microarray analysis has identified a large number of genes associated with energy metabolism, revealing that 9 genes in the glycolysis pathway are downregulated during the late larval stage with 20E pulses (White et al. 1999). During molting and pupation, high levels of 20E exhibit inhibitory effects on the mRNA levels and activity of key glycolytic enzymes, leading to downregulation of genes involved in the glycolysis pathway. At the same time, JH treatment stimulates the mRNA levels and activity of key glycolytic enzymes, possibly by antagonizing the effects of 20E (Ling et al. 2010). In this study, we observed that 14 upregulated genes were enriched in the Glycolysis/Gluconeogenesis pathway according to KEGG analysis in the EE0 vs EE50 samples. We also found several upregulated genes annotated to the electron transport chain, such as “mitochondrial respiratory chain (22)”, “respiratory chain complex (22)”, and “mitochondrial respiratory chain complex assembly (4)”. This indicates that under *s*-methoprene treatment, the energy metabolism process in *E. elutella* larvae is elevated, but it is still unclear what life activities this increased energy is utilized for.

The yellow module was selected for further analysis due to its strong correlation with (describe relevant biological traits, eg stress response, development, or other traits of interest in *E. elutella*). This module exhibited high connectivity among genes that are potentially involved in key regulatory pathways. The high number of interactions within the yellow module suggests that it could be central to important biological functions, making it an ideal candidate for identifying hub genes that may be crucial in regulating these processes. Additionally, the genes within this module demonstrated a consistent pattern across different conditions, further supporting its biological relevance. The core node genes identified in this module—Cluster-6182.18691, Cluster-6182.8343, Cluster-6182.28346, and Cluster-6182.21392—are considered hub genes due to their high connectivity within the network. Three genes (Cluster-6182.21392, Cluster-6182.8343, and Cluster-6182.28346) have been annotated through NCBI BLAST. Cluster-6182.21392 (2-acylglycerol O-acyltransferase 2-A-like) belongs to the acyltransferase family and is a key enzyme involved in lipid metabolism. It plays an important role in triglyceride synthesis, energy storage, and cell membrane structure (Cao et al. 2003). Studies have shown that *s*-methoprene can inhibit the expression of the acyltransferase gene (Du et al. 2012), which may affect lipid metabolism, inhibit triglyceride synthesis, and result in the suppression of insect metamorphosis and reproduction. Although there is currently no direct evidence linking *s*-methoprene to this gene, we hypothesize that *s*-methoprene may interfere with the activity of this gene by altering lipid metabolism, thereby affecting insect growth and development. This deserves to be further investigated. Cluster-6182.8343 (adenylyltransferase and sulfurtransferase) participates in multiple cellular functions. Adenylyltransferase may regulate ATP metabolism or cAMP signaling pathways, affecting energy supply and cell signaling in insects (Lau et al. 2009). Sulfurtransferase may be involved in detoxification metabolism or the synthesis of sulfur-containing amino acids, helping insects cope with environmental stress (Nagahara, 2011). Based on the functions of the 2 genes, we speculate that s-methoprene may affect insect energy metabolism by interfering with adenylyltransferase activity, leading to growth and development inhibition; or may inhibit sulfurtransferase activity, reducing the insect’s detoxification ability to JH analogs, thereby enhancing its toxic effects. This also deserves to be further studied. Cluster-6182.28346 (Phospholipid phosphatase 5, PLPP5) is a member of the phospholipid phosphatase family and primarily catalyzes the dephosphorylation of phosphatidic acid (PA) to form diacylglycerol (DAG), regulating lipid signaling (Zhang et al. 2017). PLPP5 regulates PA and DAG levels, affecting DAG-PKC and mTOR signaling pathways, and further regulates cell proliferation, differentiation, and apoptosis (Tang et al. 2015). In insects, PLPP5 may regulate lipid signaling pathways that influence growth and development. Based on its function, we speculate that *s*-methoprene may alter lipid signaling by inhibiting PLPP5 activity, leading to abnormal insect growth and development. For example, inhibition of PLPP5 function may lead to a reduction in DAG levels, affecting the PKC signaling pathway, thereby inhibiting cell proliferation and differentiation. Future research can use gene functional validation experiments to further explore the relationship between *s*-methoprene and PLPP5.

*s*-Methoprene exposure for 2 wk significantly affected the growth and development parameters of *E. elutella*, which increased with increasing exposure duration. The DEG annotation results indicated that *s*-methoprene has a diverse range of effects on *E. elutella* larvae, influencing critical biological processes such as hormonal regulation, signal transduction, and energy metabolism. *s*-Methoprene directly or indirectly inhibited the growth and development of *E. elutella* through these biological processes. Moreover, these biological activities revealed the molecular mechanisms by which *E. elutella* responds to *s*-methoprene exposure. Additionally, the significant control effect of *s*-methoprene on *E. elutella* larvae, combined with its environmental safety and long-lasting efficacy, underscored its tremendous potential for pest management.
